# Supplementary material for: Machine Learning Accelerates Crystallization for Structure Determination
Source: Angew Chem Int Ed Engl. 2026 May 4;65(25):e1218503. doi: 10.1002/anie.1218503 (PMC13266958; doi:10.1002/anie.1218503)

checkCIF/PLATON report

Structure factors have been supplied for datablock(s) lcz005

THIS REPORT IS FOR GUIDANCE ONLY. IF USED AS PART OF A REVIEW PROCEDURE FOR PUBLICATION, IT SHOULD NOT REPLACE THE EXPERTISE OF AN EXPERIENCED CRYSTALLOGRAPHIC REFEREE.

No syntax errors found. CIF dictionary Interpreting this report

Datablock: lcz005

Bond precision:	C-C = 0.0106 A		Wavelength=1.54184
Cell:	a=10.0208(1)	b=34.2509(3)	c=22.8960(2)
	alpha=90	beta=98.544(1)	gamma=90
Temperature:	100 K		

	Calculated	Reported
Volume	7771.19(13)	7771.19(13)
Space group	P 21/c	P 1 21/c 1
Hall group	-P 2ybc	-P 2ybc
Moiety formula	C60 H42 Ag6 F36 N12 O6	2(C15 H3 Ag3 F18 N6), 3(C10 H12 O2)
Sum formula	C60 H42 Ag6 F36 N12 O6	C60 H42 Ag6 F36 N12 O6
Mr	2358.28	2358.27
Dx,g cm-3	2.016	2.016
Z	4	4
Mu (mm-1)	13.200	13.200
F000	4560.0	4560.0
F000’	4581.45	
h,k,lmax	12,43,29	12,43,29
Nref	16617	15848
Tmin,Tmax	0.048,0.037	0.301,1.000
Tmin’	0.001	

Correction method= # Reported T Limits: Tmin=0.301 Tmax=1.000
AbsCorr = MULTI-SCAN

Data completeness= 0.954 Theta(max)= 77.963

R(reflections)= 0.0504(12918) wR2(reflections)= 0.1441(15848)

S = 1.065 Npar= 1090

The following ALERTS were generated. Each ALERT has the format
test-name_ALERT_alert-type_alert-level.
Click on the hyperlinks for more details of the test.

Alert level C

```
PLAT042_ALERT_1_C Calc. and Reported MoietyFormula Strings  Differ     Please Check
               Calc: C60 H42 Ag6 F36 N12 O6
               Rep.: 2(C15 H3 Ag3 F18 N6), 3(C10 H12 O2)
PLAT094_ALERT_2_C Ratio of Maximum / Minimum Residual Density ....       2.48 Report
PLAT213_ALERT_2_C Atom F01S            has ADP max/min Ratio .....        3.2 prolat
PLAT213_ALERT_2_C Atom F026            has ADP max/min Ratio .....        3.4 prolat
PLAT213_ALERT_2_C Atom F028            has ADP max/min Ratio .....        3.5 prolat
PLAT213_ALERT_2_C Atom F02M            has ADP max/min Ratio .....        3.3 prolat
PLAT220_ALERT_2_C NonSolvent   Resd 1  C   Ueq(max)/Ueq(min) Range        3.2 Ratio
PLAT220_ALERT_2_C NonSolvent   Resd 1  F   Ueq(max)/Ueq(min) Range        3.5 Ratio
PLAT241_ALERT_2_C High   'MainMol' Ueq as Compared to Neighbors of       O00V Check
PLAT241_ALERT_2_C High   'MainMol' Ueq as Compared to Neighbors of       O00W Check
PLAT241_ALERT_2_C High   'MainMol' Ueq as Compared to Neighbors of       O011 Check
PLAT242_ALERT_2_C Low    'MainMol' Ueq as Compared to Neighbors of       C02N Check
PLAT342_ALERT_3_C Low Bond Precision on  C-C Bonds ..............    0.01063 Ang.
PLAT360_ALERT_2_C Short  C(sp3)-C(sp3) Bond  C031     - C034     .       1.37 Ang.
PLAT906_ALERT_3_C Large K Value in the Analysis of Variance ......      2.186 Check
PLAT911_ALERT_3_C Missing FCF Refl Between Thmin & STh/L=    0.600         74 Report
                0  8  0,   1 11  0,   0 20  0,   1 20  0,   0  4  1,   0  0  2,
               -4 15  2,   9 18  2, -12  1  3, -12  2  3,   7 32  3, -12  0  4,
               -5  0  4,   1  0  4,  11  0  4, -12  1  4,  11  1  4, -12  2  4,
               11  2  4, -11  7  4, -12  1  5,  11  1  5, -12  2  5,  11  2  5,
               11  3  5,  11  4  5,  -8 30  5,  11  0  6,  11  1  6,  11  2  6,
                   (   44 More Missing: see the .ckf listing file)
PLAT971_ALERT_2_C Check Calcd Resid. Dens.  1.64Ang From C035            2.08 eA-3
PLAT971_ALERT_2_C Check Calcd Resid. Dens.  1.20Ang From C035            1.83 eA-3
PLAT971_ALERT_2_C Check Calcd Resid. Dens.  1.24Ang From C02V            1.70 eA-3
PLAT971_ALERT_2_C Check Calcd Resid. Dens.  1.58Ang From C02V            1.63 eA-3
PLAT977_ALERT_2_C Check Negative Difference Density on H03N      .      -0.46 eA-3
```

Alert level G

```
PLAT003_ALERT_2_G Number of Uiso or U(i,j)  Restrained non-H-Atoms          1 Report
PLAT004_ALERT_5_G Polymeric Structure Found with Maximum Dimension          2 Info
PLAT083_ALERT_2_G SHELXL Second Parameter in WGHT  Unusually Large      13.40 Why ?
PLAT142_ALERT_4_G s.u. on b - Axis Small or Missing .............    0.00030 Ang.
PLAT143_ALERT_4_G s.u. on c - Axis Small or Missing .............    0.00020 Ang.
PLAT186_ALERT_4_G The CIF-Embedded .res File Contains ISOR Records          1 Report
PLAT242_ALERT_2_G Low    'MainMol' Ueq as Compared to Neighbors of       C020 Check
PLAT242_ALERT_2_G Low    'MainMol' Ueq as Compared to Neighbors of       C02K Check
PLAT242_ALERT_2_G Low    'MainMol' Ueq as Compared to Neighbors of       C02L Check
PLAT242_ALERT_2_G Low    'MainMol' Ueq as Compared to Neighbors of       C02R Check
```

```
PLAT242_ALERT_2_G Low    'MainMol' Ueq as Compared to Neighbors of       C02T Check
PLAT242_ALERT_2_G Low    'MainMol' Ueq as Compared to Neighbors of       C02U Check
PLAT242_ALERT_2_G Low    'MainMol' Ueq as Compared to Neighbors of       C02X Check
PLAT242_ALERT_2_G Low    'MainMol' Ueq as Compared to Neighbors of       C02Z Check
PLAT242_ALERT_2_G Low    'MainMol' Ueq as Compared to Neighbors of       C032 Check
PLAT242_ALERT_2_G Low    'MainMol' Ueq as Compared to Neighbors of       C033 Check
PLAT242_ALERT_2_G Low    'MainMol' Ueq as Compared to Neighbors of       C037 Check
PLAT242_ALERT_2_G Low    'MainMol' Ueq as Compared to Neighbors of       C038 Check
PLAT333_ALERT_2_G Large Aver C6-Ring C-C Dist C01H     -C01V     .       1.43 Ang.
PLAT333_ALERT_2_G Large Aver C6-Ring C-C Dist C01K     -C02G     .       1.43 Ang.
PLAT335_ALERT_2_G Check Large C6 Ring C-C Range C01H     -C01V           0.16 Ang.
PLAT335_ALERT_2_G Check Large C6 Ring C-C Range C01K     -C02G           0.15 Ang.
PLAT720_ALERT_4_G Number of Unusual/Non-Standard Labels ..........        162 Note
              Ag01    Ag02    Ag03    Ag04    Ag05    Ag06    F007    O008
              F009    N00A    F00B    O00C    F00D    F00E    F00F    N00G
              N00H    F00I    F00J    N00K    F00L    N00M    F00N    N00O
              N00P    O00Q    F00R    F00S    F00T    F00U    O00V    O00W
              N00X    F00Y    N00Z    F010    O011    F012    N013    F014
              N015    F016    F017    F018    N019    F01A    F01B    F01C
              F01D    F01E    C01F    C01G    C01H    F01I    C01J    C01K
              F01L    C01M    C01N    F01O    C01P    F01Q    F01R    F01S
              C01T    H01T    C01U    C01V    H01V    C01W    H01W    C01X
              H01X    C01Y    C01Z    H01Z    C020    C021    C022    C023
              H02A    H02B    H02C    C024    H024    C025    F026    C027
              F028    C029    H029    C02A    C02B    C02C    C02D    C02E
              H02E    C02F    C02G    C02H    H02H    C02I    H02I    C02J
              C02K    C02L    F02M    C02N    C02O    H02O    C02P    C02Q
              H02Q    C02R    C02S    C02T    C02U    C02V    H02D    H02F
              H02G    F02W    C02X    C02Y    H02Y    C02Z    C030    H030
              C031    H03G    H03H    H03I    C032    C033    C034    H034
              C035    H03J    H03K    H03L    C036    H03A    H03B    H03C
              C037    C038    C039    H03D    H03E    H03F    C03A    H03P
              H03Q    H03R    C03B    H03M    H03N    H03O    C03C    H03S
              H03T    H03U
PLAT804_ALERT_5_G Number of ARU-Code Packing Problem(s) in PLATON           1 Info
PLAT860_ALERT_3_G Number of Least-Squares Restraints ............           6 Note
PLAT883_ALERT_1_G Absent Datum for _atom_sites_solution_primary ..     Please Do !
PLAT912_ALERT_4_G Missing # of FCF Reflections Above STh/L=  0.600        694 Note
PLAT913_ALERT_3_G Missing # of Very Strong Reflections in FCF ....          1 Note
                0  0  2,
PLAT941_ALERT_3_G Average HKL Measurement Multiplicity ..........         2.7 Low
PLAT969_ALERT_5_G The 'Henn et al.' R-Factor-gap value ..........       2.578 Note
              Predicted wR2: Based on SigI**2  5.59 or SHELX Weight 13.53
PLAT978_ALERT_2_G Number C-C Bonds with Positive Residual Density.          0 Info
```

---

0 **ALERT level A** = Most likely a serious problem - resolve or explain
0 **ALERT level B** = A potentially serious problem, consider carefully
21 **ALERT level C** = Check. Ensure it is not caused by an omission or oversight
31 **ALERT level G** = General information/check it is not something unexpected

2 ALERT type 1 CIF construction/syntax error, inconsistent or missing data
36 ALERT type 2 Indicator that the structure model may be wrong or deficient
6 ALERT type 3 Indicator that the structure quality may be low
5 ALERT type 4 Improvement, methodology, query or suggestion
3 ALERT type 5 Informative message, check

Validation response form

Please find below a validation response form (VRF) that can be filled in and pasted into your CIF.

```
# start Validation Reply Form
_vrf_PLAT042_lcz005
;
PROBLEM: Calc. and Reported MoietyFormula Strings  Differ     Please Check
RESPONSE: ...
;
_vrf_PLAT094_lcz005
;
PROBLEM: Ratio of Maximum / Minimum Residual Density ....       2.48 Report
RESPONSE: ...
;
_vrf_PLAT213_lcz005
;
PROBLEM: Atom F01S            has ADP max/min Ratio .....        3.2 prolat
RESPONSE: ...
;
_vrf_PLAT220_lcz005
;
PROBLEM: NonSolvent   Resd 1  C   Ueq(max)/Ueq(min) Range        3.2 Ratio
RESPONSE: ...
;
_vrf_PLAT241_lcz005
;
PROBLEM: High   'MainMol' Ueq as Compared to Neighbors of       O00V Check
RESPONSE: ...
;
_vrf_PLAT242_lcz005
;
PROBLEM: Low    'MainMol' Ueq as Compared to Neighbors of       C02N Check
RESPONSE: ...
;
_vrf_PLAT342_lcz005
;
PROBLEM: Low Bond Precision on  C-C Bonds ..............    0.01063 Ang.
RESPONSE: ...
;
_vrf_PLAT360_lcz005
;
PROBLEM: Short  C(sp3)-C(sp3) Bond  C031     - C034     .       1.37 Ang.
RESPONSE: ...
;
_vrf_PLAT906_lcz005
;
PROBLEM: Large K Value in the Analysis of Variance ......      2.186 Check
RESPONSE: ...
;
_vrf_PLAT911_lcz005
;
PROBLEM: Missing FCF Refl Between Thmin & STh/L=    0.600         74 Report
RESPONSE: ...
;
_vrf_PLAT971_lcz005
;
```

```
PROBLEM: Check Calcd Resid. Dens.  1.64Ang From C035           2.08 eA-3
RESPONSE: ...
;
_vrf_PLAT977_lcz005
;
PROBLEM: Check Negative Difference Density on H03N      .      -0.46 eA-3
RESPONSE: ...
;
# end Validation Reply Form
```

It is advisable to attempt to resolve as many as possible of the alerts in all categories. Often the minor alerts point to easily fixed oversights, errors and omissions in your CIF or refinement strategy, so attention to these fine details can be worthwhile. It is up to the individual to critically assess their own results and, if necessary, seek expert advice.

PLATON version of 17/09/2025; check.def file version of 17/09/2025

---

duplicate check

No duplication found

Datablock lcz005 - ellipsoid plot

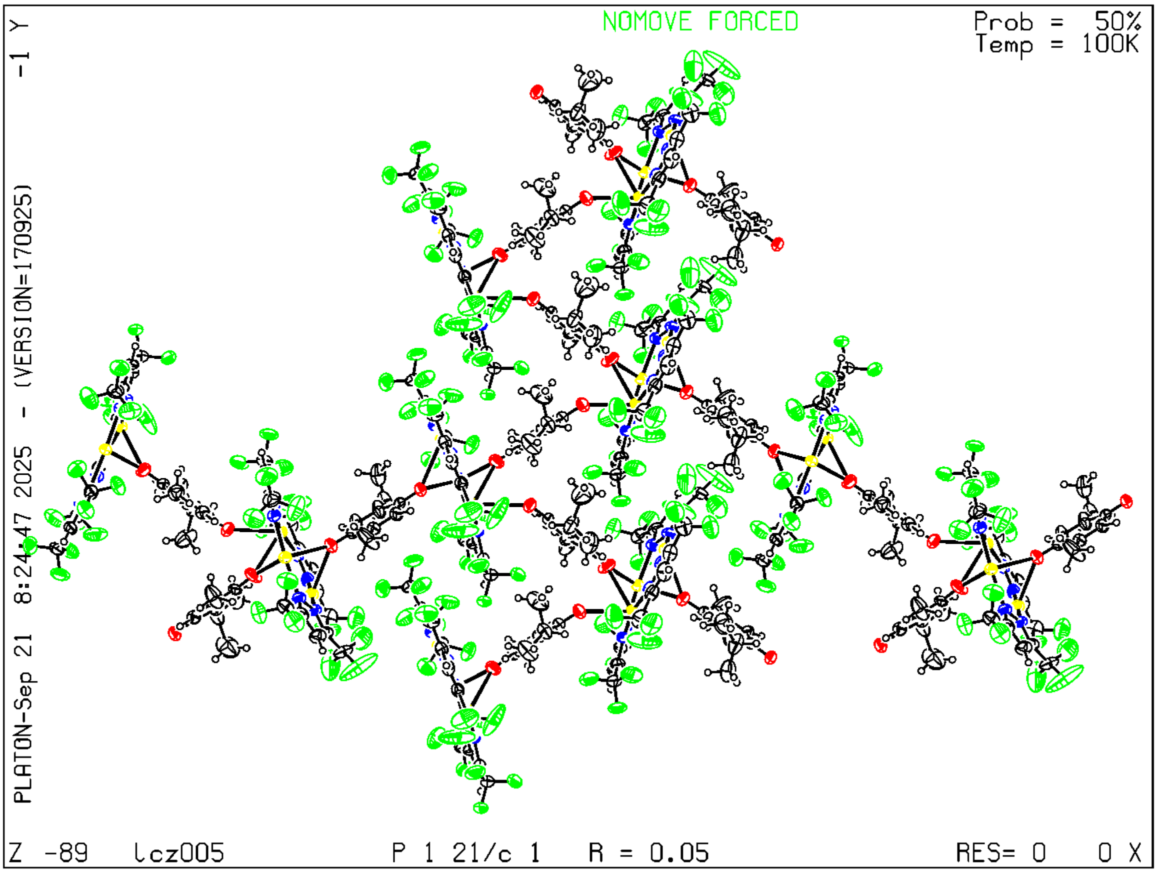

Supplement: Supplementary file 2 — Supporting File 2: anie72462‐sup‐0002‐DataFile.zip. [file ANIE-65-e1218503-s002.zip › supplementary_materials_crystallographic_data submit.zip!/cif_and_checkcif(1-114)/Ag3Pz3_5/Ag3Pz3_5_checkcif.pdf]
